# P-443. Population Pharmacokinetics (PPK) Analysis of Eravacycline (ERV) to Support Dose Selection for a Trial in Pediatric Patients Aged 8 to Less Than 18 Years with Complicated Intra-Abdominal Infection (cIAI)

**DOI:** 10.1093/ofid/ofaf695.658

**Published:** 2026-01-11

**Authors:** Claudia Jomphe, Nathalie H Gosselin, Angela Tanudra, Khurram Rana, Drew Lewis, Kajal B Larson

**Affiliations:** Certara, Montreal, Quebec, Canada; Certara, Montreal, Quebec, Canada; Innoviva Specialty Therapeutics, Inc., Waltham, MA; Innoviva Specialty Therapeutics, Madison, Connecticut; Innoviva Specialty Therapeutics, Madison, Connecticut; Entasis Therapeutics Inc., an affiliate of Innoviva Specialty Therapeutics, Inc., Waltham, MA

## Abstract

**Background:**

ERV is a synthetic, broad-spectrum fluorocycline antibiotic of the tetracycline class that is approved for the treatment of cIAI in adults. A Phase 1 study evaluated the PK, safety, and tolerability of single dose IV ERV in children 8 to < 18 years of age with suspected or confirmed bacterial infection who were receiving systemic antibiotic therapy, other than eravacycline (NCT03696550). Data from this Phase 1 trial were incorporated into a PPK model to derive dosing regimens for a Phase 2 trial in pediatric patients with cIAI (NCT06794541).

**Methods:**

A PPK model was developed using data from 12 trials of ERV in adults and the Phase 1 pediatric trial. Model-based simulations, using a virtual pediatric population (250 males and 250 females) with age-weight matched information, were conducted for two age groups: 8 to < 12 and 12 to < 18 years of age. Dosing regimens were evaluated based on their ability to yield pediatric exposures that were comparable to those observed to be safe and efficacious in adults.

**Results:**

A 3-compartment model with linear elimination adequately characterized ERV data. The model included allometric functions on clearances and on volumes of distribution, severe hepatic impairment on clearance (CL), cytochrome P450 inhibition and induction on CL and central volume of distribution (V1), and infection types on V1 (cIAI vs complicated urinary tract infection vs other infections).

The predicted steady-state exposures in cIAI pediatric patients aged 8 to < 12 years at a dose of 2 mg/kg every 12 hours (q12h) and patients aged 12 years to < 18 years at a dose of 1.5 mg/kg q12h were generally contained within the exposures in adults with cIAI.

**Conclusion:**

A PPK model was used to conduct simulations to identify ERV dosing regimens that are predicted to yield safe and efficacious exposures; these ERV dosing regimens are being evaluated in a Phase 2 clinical trial in pediatric patients with cIAI.

**Disclosures:**

Claudia Jomphe, n/a, Certara: Advisor/Consultant Angela Tanudra, MS, Innoviva Specialty Therapeutics, Inc: employee|Innoviva Specialty Therapeutics, Inc: Stocks/Bonds (Public Company) Khurram Rana, PharmD, Innoviva Specialty Therapeutics: Employee|Innoviva Specialty Therapeutics: Stocks/Bonds (Public Company) Drew Lewis, MD, MTM&H, FACP, Innoviva Specialty Therapeutics: Stocks/Bonds (Public Company) Kajal B. Larson, PhD, Innoviva Specialty Therapeutics, Inc: employee|Innoviva Specialty Therapeutics, Inc: Stocks/Bonds (Public Company)

